# Understanding Bicycle Riding Behavior and Attention on University Campuses: A Hierarchical Modeling Approach

**DOI:** 10.3390/bs15030327

**Published:** 2025-03-07

**Authors:** Wenyun Tang, Yang Tao, Jiayu Gu, Jiahui Chen, Chaoying Yin

**Affiliations:** 1 College of Automobile and Traffic Engineering, Nanjing Forestry University, Nanjing 210037, China; tangwy@njfu.edu.cn (W.T.); taoyang@njfu.edu.cn (Y.T.); 3241600709@njfu.edu.cn (J.C.); 2 Civil Aviation College, Nanjing University of Aeronautics and Astronautics, Nanjing 211106, China; sx2207072@nuaa.edu.cn

**Keywords:** bicycle riding behavior, attention, university campuses, hierarchical modeling approach

## Abstract

The traffic behavior characteristics within university campuses have received limited scholarly attention, despite their distinct differences from external road networks. These differences include the predominance of non-motorized vehicles and pedestrians in traffic flow composition, as well as traffic peaks primarily coinciding with class transition periods. To investigate the riding behavior of cyclists on university campuses, this study examines cyclist attention, proposes a novel method for constructing a rider attention recognition framework, utilizes a hierarchical ordered logistic model to analyze the factors influencing attention, and evaluates the model’s performance. The findings reveal that traffic density and riding style significantly influence cyclists’ eye-tracking characteristics, which serve as indicators of their attention levels. The covariates of lane gaze time and the coefficient of variation in pupil diameter exhibited significant effects, indicating that a hierarchical ordered logistic model incorporating these covariates can more effectively capture the impact of influencing factors on cyclist attention. Moreover, the hierarchical ordered logistic model achieved a 7.22% improvement in predictive performance compared to the standard ordered logistic model. Additionally, cyclists exhibiting a “conservative” riding style were found to be more attentive than those adopting a “aggressive” riding style. Similarly, cyclists navigating “sparse” traffic conditions were more likely to maintain attention compared to those in “dense” traffic scenarios. These findings provide valuable insights into the riding behavior of university campus cyclists and have significant implications for improving traffic safety within such environments.

## 1. Introduction

In recent years, the expansion of university campuses has led to increasingly complex road networks and traffic structures. However, outdated traffic management practices and ambiguous road-use rights have resulted in significant pedestrian–vehicle interactions and frequent conflicts, adversely affecting the daily activities of students and staff (Shi & Ma, 2018). Campus traffic is characterized by convergence around major facilities such as teaching buildings, libraries, and cafeterias, with periodic peaks corresponding to class schedules. Although motorized vehicles are not entirely excluded, the enclosed nature of campuses and the associated safety considerations make low-speed transportation more appropriate (Balsas, 2003). Non-motorized vehicles, especially bicycles, which serve as a primary mode of campus transportation, have increased in prevalence alongside campus expansion. The interactions among bicycles and between these bicycles and pedestrians constitute a significant source of traffic conflicts, undermining traffic efficiency and posing substantial safety risks for campus users.

Traffic safety within university campuses has become an increasingly pressing concern. Several studies have utilized micro-simulation and modeling approaches to examine traffic conditions and conflicts across various scenarios, aiming to evaluate the safety of campus travel (Fernandes et al., 2023; Malayjerdi et al., 2021; Tomás et al., 2021). In addition, some studies have explored the psychological and behavioral characteristics of campus drivers, uncovering the relationships between driver attitudes, traffic congestion, and accidents and subsequently proposing practical traffic management strategies (Hashim et al., 2020). Research on campus infrastructure and its influence on traffic safety also holds considerable practical significance, addressing elements such as traffic signage (Clack et al., 2000) and lighting conditions (Feyli & Abadi, 2024). Nevertheless, most existing research on campus safety predominantly focuses on motorized vehicles and pedestrians, with relatively limited attention given to bicycle safety. On campuses with high traffic volumes and diverse traffic compositions, cyclists are subject to varying levels of psychological stress due to the presence of dense mixed traffic, which can heighten the risk of accidents (Sanders, 2015). Furthermore, excessive campus traffic volumes can result in accidents that lead to localized congestion or even secondary collisions, thereby compounding safety risks.

The relationship between driver attention and traffic safety is profoundly interconnected. Identifying the physiological characteristics of drivers has emerged as an effective method for assessing attention levels. With advancements in technology, growing research efforts have employed monitoring and recognition techniques to link drivers’ physiological attributes to their attention levels, facilitating objective and accurate assessments during driving. For example, Ruscio et al. (2017) evaluated drivers’ attention by monitoring heart rate variability regulated by the nervous system. Other studies have investigated the relationship between alcohol consumption and attention during simulated driving through the analysis of auditory features (Banz et al., 2022). Additionally, the dynamic visual characteristics of drivers are closely associated with traffic accidents. The road environment stimulates visual perception, influencing drivers’ psychological, physiological, behavioral, and fatigue states. Metrics derived from eye-tracking, such as blink frequency, fixation and saccade durations, and pupil diameter, have been widely validated as reliable indicators of driver attention under various conditions, including normal states (Shen et al., 2021; Li et al., 2020), intoxication (De Blasiis et al., 2020), fatigue (Wang et al., 2016), and the effects of psychoactive substances (Shahidi Zandi et al., 2021). However, most existing research on attention focuses primarily on motor vehicle drivers, with relatively limited attention directed toward bicyclists. Nevertheless, the approach of examining driving behavior through drivers’ eye movement characteristics can be extended to studying the riding behavior of bicyclists.

Building on existing research, further investigations into the attention of bicycle riders through diverse measurement methods hold considerable academic significance. This is particularly pertinent in the context of complex and dynamic campus traffic environments, where the attention and response characteristics of bicycle riders may differ markedly from those of motor vehicle drivers. Compared to motor vehicle operation, bicycle riding is subject to a broader range of environmental influences, including narrow roadways, dense pedestrian traffic, and mixed vehicle flows (Lei et al., 2024). These conditions can heighten distraction and psychological stress, thereby impacting riders’ risk perception and decision-making behavior. The integration of advanced technologies such as eye-tracking provides a more comprehensive and objective approach to examining the visual search strategies and information processing patterns of bicycle riders within campus road networks. Such analysis offers valuable insights into their interactions with the surrounding traffic environment and contributes to the development of targeted measures to enhance traffic safety.

Building on the preceding analysis, this study aims to investigate the attention of bicyclists within a university campus setting. Eye-tracking technology was employed to collect eye movement data while cycling under various road conditions. A hierarchical ordered logistic regression model was developed to assess cyclists’ attention, identify factors influencing their attention, and compare the findings with those derived from a standard ordered logistic regression model.

The remainder of this paper is structured as follows: Section 2 outlines the experimental design, Section 3 details the model construction, Section 4 presents the results and analysis, and Section 5 concludes the study.

## 2. Experimental Design

### 2.1. Experimental Preparation

#### 2.1.1. Experimental Scenario

In this study, Sections A, B, and C of the main roads within a university campus were selected as experimental sites for research. None of these road sections contain intersections, and the cross-sectional configurations are consistent across all three. The selected sections are linear roads with widths of approximately 7 m and lengths of 104 m, 90 m, and 120 m, respectively. The gradients for Sections A and B are 7.9% and 4.4%, respectively, while Section C is a level road. To eliminate potential interference, motor vehicles were prohibited from accessing these sections during the experiments.

The traffic flow on campus roads exhibits considerable temporal variability due to students’ daily activities, such as attending classes, dining, and other routines. The experimental sections are located along main roads frequently used by students for commuting between classes. Video-based surveys revealed significant variations in pedestrian and bicycle flow densities across different time periods. High flow densities were observed during peak periods, such as 7:00–8:00 a.m. and 12:00–2:00 p.m., whereas lower densities were recorded during periods such as 8:00–9:00 a.m. Flow densities during other daytime periods were relatively moderate.

#### 2.1.2. Experimental Equipment and Participants

**Figure 1:**
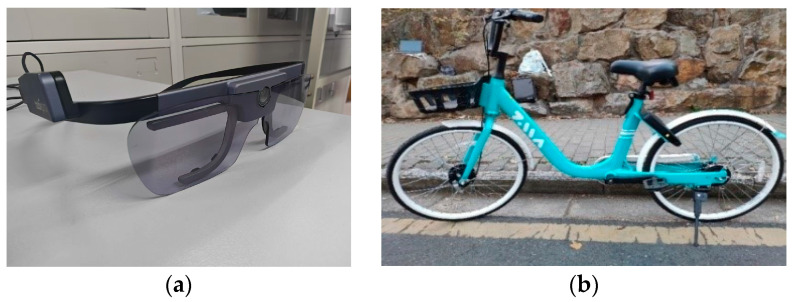
Experimental equipment. (**a**) Eye tracker; (**b**) experimental bicycle.

In this experiment, the Tobii Glass2 wearable eye tracker was utilized to record visual parameters of riders’ eye movement behaviors, including fixation, saccades, and pupil diameter. The device operated at a sampling frequency of 50 Hz, with single-point calibration, and data were recorded in a remote offline mode. The experimental vehicle was a shared bicycle in normal operating condition, with the brakes, tires, seat height, and handlebars adjusted to ensure optimal performance throughout the experiment. Figure 1 shows the eye tracker and the experimental bicycle.

A total of 24 participants were recruited for the study, consisting of 12 males and 12 females, aged 18 to 24 years (mean age, 20.08 years; standard deviation, 2.15). All participants were proficient in riding non-motorized vehicles and familiar with the experimental route. They exhibited normal visual function (corrected vision above 1.0), good riding habits (assessed during recruitment to exclude behaviors such as phone use or one-handed riding), and no physiological impairments. Prior to the experiment, participants were instructed to ensure adequate rest, avoid consuming stimulating foods, and refrain from activities causing excessive fatigue or emotional distress. Additionally, we informed all participants of the ethical review results of the experiment. We confirmed that all data would be anonymized to protect the privacy rights of the participants.

#### 2.1.3. Experimental Procedure

Prior to the experiment, it was imperative that we ensured that participants were thoroughly familiar with the designated cycling route. Participants were instructed to ride according to their personal experience and habits and the prevailing traffic conditions. Following this, the eye-tracking device was calibrated, and participants underwent a monitoring training session to confirm that the equipment did not interfere with their riding performance. During the formal testing phase, the eye-tracking device was operated via a computer platform. Recording began as the participant commenced the cycling task. Each participant rode from the starting point to the endpoint to complete the designated route test and then returned to the starting point. Upon completion, the eye-tracking device reconnected with the computer software, and the recording was terminated, marking the conclusion of one experimental trial. This procedure was repeated until all participants had completed tests.

### 2.2. Data Acquisition and Processing

A total of 24 volunteers were initially recruited to participate in the experiment. However, during the equipment calibration phase, 15 volunteers encountered difficulties maintaining a smooth focus, resulting in a final sample size of 9 participants. During the experiment, each participant was required to cycle once in both directions across the three test sections during peak and off-peak hours. Consequently, the total raw dataset comprised 108 sets of data. Due to factors such as signal interruptions and data transmission errors, 97 datasets were ultimately deemed valid for analysis. While the number of participants was limited to nine, the eye movement data collected from the 97 valid datasets were sufficient for the analysis, as this study does not emphasize the socio-demographic attributes of participants.

**Figure 2:**
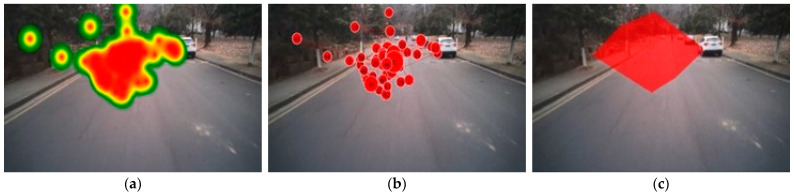
Participants’ fixation characteristic maps. (**a**) Fixation heatmaps; (**b**) fixation trajectory; (**c**) fixation area.

The experimental data were analyzed using the Ergo-LAB Human–Machine Environment Synchronization Cloud Platform. Various eye-tracking metrics were extracted, including fixation heatmaps, the fixation trajectory, and the fixation area, as depicted in Figure 2.

## 3. Model Construction

### 3.1. Indicator Selection

The ErgoLAB platform generates a comprehensive set of eye-tracking metrics for each participant in every experimental trial. These metrics include the fixation time percentage, saccadic time percentage, average fixation time, average saccadic time, average blink time, blink frequency per second/minute, average pupil diameter, maximum/minimum pupil diameter, and pupil diameter standard deviation, as well as the distribution of fixation points, fixation time distributions, and average fixation time parameters for each region of interest. The data from each trial were organized and analyzed to compute averages, standard deviations, and other relevant statistics. Among these indicators, the fixation time percentage, saccadic time percentage, blink frequency per minute, average pupil diameter, average fixation time, average saccadic time, and fixation time distribution were identified as critical measures for reflecting the participants’ attention levels from various perspectives, such as attention allocation (Shan & Jiao, 2021). These indicators were consequently selected as the primary data sources for subsequent analysis.

In the model constructed for this study, the dependent variable is the cyclist’s level of attention. Fixation behavior, saccadic behavior, and other eye movements were utilized as proxies for attention levels. Indicators such as the fixation time percentage, saccadic time percentage, and average blink time each contribute unique insights into attention. Principal component analysis (PCA) was applied to reduce the dimensionality of the 97 sets of eye-tracking data. A composite score representing the cyclist’s overall attention level was then derived based on the eigenvalues of the selected indicators. Based on this, cyclists’ attention scores were categorized into three distinct intervals, representing varying attention levels: “Focused”, “Scattered”, and “Distracted”.

#### 3.1.1. Independent Variable

A variety of factors may influence bicyclists’ attention. However, due to experimental limitations, the selection of road environment parameters was initially constrained to slope, road width, and the length of the experimental sections. Among these, the primary variation across the three sections was the slope. Nonetheless, a one-way ANOVA revealed no significant differences in attention related to the longitudinal slope among participants, suggesting that this factor may have a limited impact in the relatively simple and uniform road environment of a university campus. As a result, slope was excluded as a dependent variable in further analyses.

One-way ANOVA identified road traffic flow and riding style as significant factors influencing bicyclists’ attention. Accordingly, traffic density and riding style were selected as independent variables for the model. Due to the influence of student class schedules, pedestrian and non-motorized vehicle traffic flows on the surveyed routes exhibited a clear tidal pattern. Traffic density was therefore classified into three categories: “Sparse”, “Moderate”, and “Dense”, based on the time periods during the survey. Riding style was categorized based on relative cycling speeds for comparison with other subjects, with two classifications: “Aggressive” and “Conservative”. Riders were categorized as “Aggressive” if their average speed exceeded the mean speed of all subjects for the specific scenario; otherwise, they were categorized as “Conservative”. While road slope may have influenced cyclists’ speed to some extent, the classification of riding style was determined using the overall average speed of all participants as the reference. Among the nine participants, four were classified as exhibiting an “Aggressive” riding style, while the remaining five were categorized as “Conservative”.

#### 3.1.2. Covariates

The hierarchical ordered logistic model was employed to analyze variations in cyclists’ attention under the influence of distinct factors. To improve the model’s applicability and ensure robust analysis, covariates were introduced as control variables. Two eye-tracking indicators were selected as covariates for the model.

1. Lane fixation time: The lane fixation time represents the intensity of a participant’s gaze concentration. This metric is calculated by summing the fixation time across regions and categorizing it into three areas: the driving lane area, the left side area, and the right side area. The proportion of fixation time allocated to the driving lane area out of the total fixation time is defined as the lane fixation time (Shan & Jiao, 2021). As a continuous variable, this indicator allows inference about whether the participant’s attention is focused on the driving lane, indicating engagement with the primary cycling task.
2. Pupil diameter coefficient of variation: Variations in pupil diameter due to external environmental factors provide insights into a participant’s cognitive workload (Ke et al., 2021). An increase in pupil diameter often signifies greater effort directed toward attentional focus on a target. The pupil diameter coefficient of variation (P) is calculated as the ratio of the standard deviation of the participant’s pupil diameter to their average pupil diameter during the experiment. This metric serves as an indicator of attention (Train, 2009). Formula (1) is for calculating *P*.

(1) $$P=\frac{\sigma }{\bar{d}},$$

In this equation, *P* represents the pupil diameter coefficient of variation, σ is the standard deviation of the pupil diameter, and $\bar{d}$ is the average pupil diameter of the participants.

### 3.2. Hierarchical Ordinal Logistic Model

In the assessment of driver attention and related research, employed modeling methods include Support Vector Machines (Becerra-Sánchez et al., 2019) and Convolutional Neural Networks (Smoliński et al., 2024), among others. However, the majority of these approaches overlook the influence of individual differences. To address this gap, the present study adopts a hierarchical ordinal logistic model grounded in logistic regression, which accounts for individual variability, and constructs a standard ordinal logistic model for comparative analysis.

The modeling procedure for the hierarchical ordinal logistic model parallels that of the conventional ordinal logistic model, with the primary distinction being the incorporation of variability in threshold differences among drivers. In the hierarchical model, thresholds are calculated based on eye-tracking metrics specific to each individual driver. Consequently, this study focuses exclusively on a detailed exposition of the modeling process for the hierarchical ordinal logistic model. By design, the hierarchical ordinal logistic model captures the heterogeneity in attention thresholds among participants. Specifically, the threshold parameters are linked to individual eye-tracking metrics, providing a tailored representation of cyclist variability.

Let $F_{ij}$ represent the attention level of the *j*-th cyclist on the *i*-th segment of the route (where i=1,2,3; j=1,2⋯⋯9, corresponding to the following attention levels: 1 (Attention level: Distracted), 2 (Attention level: Scattered), and 3 (Attention level: Focused)). This model assumes the existence of a continuous latent variable $F_{ij}^{*}$ associated with the discrete variable $F_{ij}$. The process of obtaining the discrete values of $F_{ij}$ involves establishing multiple thresholds and intervals of $F_{ij}^{*}$. Let $\gamma_{kj}$ represent the attention level thresholds for the *j*-th cyclist. The expression for the hierarchical ordinal logistic model is as follows:

(2) $$F_{ij}=\left\{\begin{array}{l} 1,\;-\infty <F_{ij}^{*}<\gamma_{1j}\\ 2,\;\;\gamma_{1j}<F_{ij}^{*}<\gamma_{2j}\\ 3,\;\;\gamma_{2j}<F_{ij}^{*}<+\infty \end{array}\right.,$$

It can be written in the following form, similar to a conventional linear regression model:

(3) $$F_{ij}=\theta_{ij}+\varepsilon_{ij},$$

(4) $$\theta_{ij}=\sum_{p=1}^{p}\beta_{ij}x_{pij},$$

In this equation, $x_{pij}$ represents the *p*-th covariate of the *j*-th cyclist on the *i*-th segment, and $\beta_{ij}$ is the regression coefficient; $\theta_{ij}$ is the observed utility term; and $\varepsilon_{ij}$ is the error term, which follows a double exponential distribution.

The cumulative distribution function for $F_{ij}$ is given by

(5) $$P_{ij\left(k\right)}=\mathrm{Pr}\left(F_{ij}<k\right)=F\left(\gamma_{ij}-\theta_{ij}\right)=\frac{\exp\left(\gamma_{ki}-\theta_{ij}\right)}{1+\exp\left(\gamma_{ki}-\theta_{ij}\right)},$$

Perform the logistic transformation on the above cumulative distribution function:

(6) $$Logit\left(P_{ij\left(k\right)}\right)=\log\left(\frac{P_{ij\left(k\right)}}{1-P_{ij\left(k\right)}}\right)=\log\left(\frac{\mathrm{Pr}\left(F_{ij}\leq k\right)}{\mathrm{Pr}\left(F_{ij}>k\right)}\right)=\gamma_{kj}-\theta_{ij},$$

For an individual cyclist, $\gamma_{kj}$ is used to represent the attention thresholds of different cyclists:

(7) $$\gamma_{kj}=\gamma_{k}+\sum_{q=1}^{Q}\alpha_{q}z_{qj}+B_{j},$$

In this equation, the intercept $\gamma_{k}$ represents the constant component of the attention thresholds across all cyclists; $z_{qj}$ denotes the covariates accounting for differences among cyclists, introducing variability in thresholds across individuals; $\alpha_{q}$ indicates the impact of the *q*-th cyclist-specific variable on the attention thresholds; and $B_{j}$ is a random variable with a mean of zero, following a normal distribution tau~Gamma(0.01,0.01).

## 4. Results and Analysis

### 4.1. Parameter Estimation

Table 1 and Table 2 present the parameter estimation results derived from the ordered logistic model and the hierarchical ordered logistic model, respectively. A comparison of the parameter estimates from the two models reveals that the effects of the two independent variables, riding style and traffic density, are largely consistent. In both models, riding style emerges as a significant influencing factor, and the smallest traffic density, relative to the largest traffic density, has a significant impact on cyclists’ attention levels. The analysis results indicate that, regarding riding style, conservative riders exhibit higher levels of attention compared to aggressive riders. With respect to traffic density, cyclists demonstrate greater attention when the traffic volume is lower. This finding may be attributed to the higher riding speeds associated with lower traffic volumes (Castro et al., 2025), which could heighten cyclists’ concerns about unexpected intrusions by vehicles or pedestrians into their riding path. Although a high traffic volume on regular roads typically indicates more complex traffic conditions, the situation differs on university campuses. During peak hours—such as when students are commuting to and from class—large numbers of individuals are concentrated in specific locations, including classrooms, dormitories, cafeterias, and school gates, following relatively fixed routes. Notably, the movement of vehicles and pedestrians tends to be in the same general direction. As a result, rather than increasing traffic complexity, high traffic volumes on campus may contribute to more predictable and orderly traffic conditions. Conversely, when the traffic volume is low, cyclists may face heightened challenges due to factors such as road alignment, obstructed sight distances, vehicle speeding, and pedestrian crossings. These elements can introduce unexpected events that are beyond the cyclists’ control, thereby increasing the complexity of the traffic environment. Furthermore, the results in Table 2 highlight that the two covariates—the lane gaze time and the coefficient of variation for pupil diameter—directly influence the thresholds used to classify attention, and both exhibit statistically significant effects. This suggests that incorporating covariates and employing the hierarchical ordered logistic model provide a more effective framework for capturing the factors influencing cyclists’ attention levels.

### 4.2. Model Evaluation and OR Analysis

#### 4.2.1. Model Evaluation

This study compares and evaluates the predictive accuracy of the two models. A prediction is deemed accurate when the predicted cyclist attention level aligns with the attention level determined from eye movement metrics; otherwise, it is considered inaccurate. According to the prediction accuracy results presented in Table 3, the hierarchical ordered logistic model achieves an overall prediction accuracy of 53.61%, whereas the ordered logistic model attains a prediction accuracy of 46.39%. The hierarchical ordered logistic model demonstrates superior performance, with an improvement of 7.22 percentage points in accuracy compared to the standard ordered logistic model.

#### 4.2.2. Odds Ratio (OR) Analysis

Based on the results of the OR analysis, for “riding style”, using “aggressive” as the reference, the OR value for “riding style” is 3.105. Under otherwise identical conditions, cyclists with a “conservative” riding styles are more likely to maintain attention compared to “aggressive” cyclists. For “traffic density”, using “dense” traffic as the reference, cyclists are more likely to maintain attention under “sparse” traffic, with an OR value of 7.622. Similarly, cyclists are more likely to maintain attention under “moderate” traffic than under “dense” traffic, with an OR value of 2.368. When recoding traffic density and taking “sparse” traffic as the reference, the regression results indicate that cyclists are less likely to maintain attention under “moderate” traffic compared to “sparse” traffic, with an OR value of 0.310.

## 5. Conclusions

This study employed the Tobii Glasses 2 wearable eye tracker to collect visual eye movement data from cyclists under various road conditions, including differing slopes and traffic densities, resulting in 97 valid datasets. From these data, indicators related to fixation, saccade, and pupil diameter were calculated. Principal component analysis (PCA) was utilized to process these indicators and derive a composite score representing cyclists’ attention levels. Using cyclists’ attention levels as the dependent variable, and traffic density and riding style as independent variables, a hierarchical ordered logistic regression model was developed to examine three distinct levels of attention. For comparison, an ordered logistic regression model that did not incorporate individual differences was also constructed.

The results reveal that both riding style and traffic density significantly influence riders’ attention levels. Specifically, with regard to riding style, conservative riders exhibit higher attention compared to aggressive riders. In terms of traffic density, cyclists demonstrate greater attention under low-traffic conditions. This finding may be attributed to the higher riding speeds associated with lower traffic volumes, which heighten concerns about unexpected intrusions by vehicles or pedestrians into the cycling path. This conclusion may diverge from findings related to the attention of cyclists on regular roads, highlighting a key distinction between traffic behavior on university campuses and that on external roads. This suggests that traffic dynamics on campuses warrant further investigation and comparison. Future studies should thus engage in a more nuanced examination, rather than directly extrapolating conclusions drawn from regular road settings to the context of university campus traffic behavior. Additionally, the two covariates—lane gaze time and the coefficient of variation for pupil diameter—directly affect the thresholds used to classify attention, and both demonstrate statistically significant effects. This underscores the importance of incorporating covariates and highlights the hierarchical ordered logistic model’s capacity to more effectively capture the factors influencing cyclists’ attention levels. Furthermore, the hierarchical ordered logistic model achieves a prediction accuracy that is 7.22% higher than that of the standard ordered logistic model.

The findings of this study hold both theoretical and practical significance for enhancing cyclists’ attention. However, the ordered logistic model used for comparison was structurally similar to the hierarchical ordered logistic model. Future research could explore alternative modeling approaches, such as Artificial Neural Networks (ANNs), for comparative analysis. This study primarily examined the cycling attention levels of university students, with a relatively limited dataset. Furthermore, the road environments on university campuses are comparatively simpler than those of external road networks. Future research could address these limitations by increasing the sample size, incorporating a broader range of participant demographics, and investigating cyclists’ attention levels under more diverse road conditions to obtain more comprehensive insights. Moreover, attention levels associated with other modes of campus transportation—such as scooters, e-bikes, and skateboards—warrant further exploration. Future studies could consider integrating additional variables, such as speed variability or lateral positioning, into the classification of riding styles. The methodology and conclusions presented in this study provide a valuable foundation for future research, whether that involves investigating other scenarios or other types of road participants or using alternative parameters to study attention levels.

## Figures and Tables

**Table 1:** Estimated parameters of the ordered logistic model.

						95% Confidence Interval	
	Estimation	Standard Deviation	Wald	df	Sig	Upper Limit	Lower Limit
Attention level = 0	0.688	0.500	1.895	1	0.169	−0.292	1.668
Attention level = 1	2.337	0.554	17.826	1	0.000	1.252	3.423
Attention level = 2	0						
Riding Style = 0	1.234	0.402	9.417	1	0.002 ***	0.446	2.022
Riding Style = 1	0			0			
Traffic density = 1	1.792	0.591	9.210	1	0.002 ***	0.635	2.950
Traffic density = 2	0.787	0.547	2.070	1	0.150	−0.285	1.860
Traffic density = 3	0			0			

***: Significant at 99% confidence level.

**Table 2:** Estimated parameters of the hierarchical ordered logistic model.

						95% Confidence Interval	
	Estimation	Standard Deviation	Wald	df	Sig	Upper Limit	Lower Limit
Attention level = 0	10.281	3.880	7.022	1	0.008	2.677	17.885
Attention level = 1	12.119	3.935	9.484	1	0.002	4.406	19.833
Attention level = 2	0						
Riding Style = 0	1.133	0.414	7.488	1	0.006 ***	0.321	1.944
Riding Style = 1	0			0			
Traffic density = 1	2.031	0.618	10.800	1	0.001 ***	0.820	3.243
Traffic density = 2	0.862	0.566	2.314	1	0.128	−0.248	1.972
Traffic density = 3	0			0			
Fixation	0.105	0.040	6.730	1	0.009 ***	0.026	0.184
Pupil	−9.560	4.778	4.004	1	0.045 **	−18.924	−0.196

***: Significant at 99% confidence level. **: Significant at 95% confidence level.

**Table 3:** Model prediction accuracy.

Cross Table		Predicted Response Categories							
		Ordered Logistic Model				Hierarchical Ordered Logistic Model			
		Distracted	Scattered	Focused	Total	Distracted	Scattered	Focused	Total
Attention level	Distracted	18	6	7	31	20	7	4	31
	Scattered	12	7	13	32	9	11	12	32
	Focused	5	9	20	34	2	11	21	34
	Total	35	22	40	97	31	29	37	97
Predictive accuracy		46.39%				53.61%			

## Data Availability

The data presented in this study are openly available in FigShare at 10.6084/m9.figshare.27902439.V1.

## References

[B1-behavsci-15-00327] Balsas C. J. L. (2003). Sustainable transportation planning on college campuses. Transport Policy.

[B2-behavsci-15-00327] Banz B. C., Camenga D. R., Crowley M. J., Vaca F. E. (2022). Binge drinking and alcohol-related symptoms may underlie patterns of dynamic brain oscillations of resource allocation during high-fidelity driving simulation. Traffic Injury Prevention.

[B3-behavsci-15-00327] Becerra-Sánchez E. P., Reyes-Muñoz A., Guerrero-Ibáñez J. A. (2019). Wearable sensors for evaluating driver drowsiness and high stress. IEEE Latin America Transactions.

[B4-behavsci-15-00327] Castro G. P., Fredrik J., Johan O. (2025). Empirical study of bicycle traffic characteristics relevant for microscopic simulation. Journal of Cycling and Micromobility Research.

[B5-behavsci-15-00327] Clack Z. A., Pitts S. R., Kellermann A. L. (2000). Do reminder signs promote use of safety belts?. Annals of Emergency Medicine.

[B6-behavsci-15-00327] De Blasiis M. R., Ferrante C., Veraldi V., Arezes P. (2020). Driving risk assessment under the effect of alcohol through an eye tracking system in virtual reality. Advances in safety management and human factors. AHFE 2019. Advances in intelligent systems and computing.

[B7-behavsci-15-00327] Fernandes P., Macedo E., Tomás R., Coelho M. C. (2023). Hybrid electric vehicle data-driven insights on hot-stabilized exhaust emissions and driving volatility. International Journal of Sustainable Transportation.

[B8-behavsci-15-00327] Feyli N., Abadi M. G., Wei H. (2024). Subjective walkability and bikeability: Analysis of the built environment and safety at a campus area. Proceedings of the international conference on transportation and development 2024: Transportation planning, operations, and transit (ICTD 2024), Atlanta, Georgia, 15–18 June 2024.

[B9-behavsci-15-00327] Hashim R., Masuri M. G., Md Isa K. A., Ghazali A. R. (2020). Driversâ€™ Attitudes on Campus Roads: A review. Environment-Behaviour Proceedings Journal.

[B10-behavsci-15-00327] Ke Q., Ding S., Qin Q. (2021). Health information readability affects users’ cognitive load and information processing: An eye-tracking study. Data Analysis and Knowledge Discovery.

[B11-behavsci-15-00327] Lei X., Tu P., Zhang J., Zhang Y. (2024). Design of urban non-motorized lane width under mixed traffic of electric bicycles. Journal of Beijing University of Technology.

[B12-behavsci-15-00327] Li X., Schroeter R., Rakotonirainy A., Kuo J., Lenné M. G. (2020). Effects of different non-driving-related-task display modes on drivers’ eye-movement patterns during take-over in an automated vehicle. Transportation Research Part F: Traffic Psychology and Behaviour.

[B13-behavsci-15-00327] Malayjerdi M., Baykara B. C., Sell R., Malayjerdi E. (2021). Autonomous vehicle safety evaluation through a high-fidelity simulation approach. Proceedings of the Estonian Academy of Sciences.

[B14-behavsci-15-00327] Ruscio D., Bos A. J., Ciceri M. R. (2017). Distraction or cognitive overload? Using modulations of the autonomic nervous system to discriminate the possible negative effects of advanced assistance systems. Accident Analysis & Prevention.

[B15-behavsci-15-00327] Sanders R. L. (2015). Perceived traffic risk for cyclists: The impact of near miss and collision experiences. Accident Analysis & Prevention.

[B16-behavsci-15-00327] Shahidi Zandi A., Comeau F. J. E., Mann R. E., Di Ciano P., Arslan E. P., Murphy T., Le Foll B., Wickens C. M. (2021). Preliminary eye-tracking data as a nonintrusive marker for blood Δ-9-tetrahydrocannabinol concentration and drugged driving. Cannabis and Cannabinoid Research.

[B17-behavsci-15-00327] Shan X. L., Jiao P. P. (2021). Study on influence of roadside billboard on drivers’ attention based on eye tracking characteristics. Journal of Highway and Transportation Research and Development.

[B18-behavsci-15-00327] Shen N., Zhu Y., Oroni C. Z., Wang L. (2021). Analysis of drivers’ eye movements with different experience and genders in straight-line driving. 2021 IEEE international conference on dependable, autonomic and secure computing, international conference on pervasive intelligence and computing, international conference on cloud and big data computing, intl conf on cyber science and technology congress (DASC/PiCom/CBDCom/CyberSciTech), virtual, 25–28 October 2021.

[B19-behavsci-15-00327] Shi J., Ma K., Stanton N. (2018). Digital touchpoints in campus slow traffic service system. Advances in human aspects of transportation. AHFE 2017. Advances in intelligent systems and computing.

[B20-behavsci-15-00327] Smoliński A., Forczmański P., Nowosielski A. (2024). Processing and integration of multimodal image data supporting the detection of behaviors related to reduced concentration level of motor vehicle users. Electronics.

[B21-behavsci-15-00327] Tomás R., Fernandes P., Macedo E., Coelho M. C. (2021). Carpooling as an immediate strategy to post-lockdown mobility: A case study in university campuses. Sustainability.

[B22-behavsci-15-00327] Train K. E. (2009). Discrete choice methods with simulation.

[B23-behavsci-15-00327] Wang M. S., Jeong N. T., Kim K. S., Choi S. B., Yang S. M., You S. H., Lee J. H., Suh M. W. (2016). Drowsy behavior detection based on driving information. International Journal of Automotive Technology.

